# Anti-Inflammatory and Cytoprotective Effects of TMC-256C1 from Marine-Derived Fungus *Aspergillus* sp. SF-6354 via up-Regulation of Heme Oxygenase-1 in Murine Hippocampal and Microglial Cell Lines

**DOI:** 10.3390/ijms17040529

**Published:** 2016-04-08

**Authors:** Dong-Cheol Kim, Kwang-Ho Cho, Wonmin Ko, Chi-Su Yoon, Jae Hak Sohn, Joung Han Yim, Youn-Chul Kim, Hyuncheol Oh

**Affiliations:** 1 Institute of Pharmaceutical Research and Development, College of Pharmacy, Wonkwang University, Iksan 54538, Korea; kimman07@hanmail.net (D.-C.K.); zxzxguild@gmail.com (K.-H.C.); rabis815@naver.com (W.K.); ycs1991@naver.com (C.-S.Y.); yckim@wku.ac.kr (Y.-C.K.); 2 College of Medical and Life Sciences, Silla University, Busan 46958, Korea; jhsohn@silla.ac.kr; 3 Korea Polar Research Institute, KORDI, Yeonsu-gu, Incheon 21990, Korea; jhyim@kopri.re.kr

**Keywords:** TMC-256C1, *Aspergillus*, marine fungus, neuroprotective effect, anti-neuroinflammatory effect, heme oxygenase-1 (HO-1)

## Abstract

In the course of searching for bioactive secondary metabolites from marine fungi, TMC-256C1 was isolated from an ethyl acetate extract of the marine-derived fungus *Aspergillus* sp. SF6354. TMC-256C1 displayed anti-neuroinflammatory effect in BV2 microglial cells induced by lipopolysaccharides (LPS) as well as neuroprotective effect against glutamate-stimulated neurotoxicity in mouse hippocampal HT22 cells. TMC-256C1 was shown to develop a cellular resistance to oxidative damage caused by glutamate-induced cytotoxicity and reactive oxygen species (ROS) generation in HT22 cells, and suppress the inflammation process in LPS-stimulated BV2 cells. Furthermore, the neuroprotective and anti-neuroinflammatory activities of TMC-256C1 were associated with upregulated expression of heme oxygenase (HO)-1 and nuclear translocation of nuclear factor-E2-related factor 2 (Nrf2) in HT22 and BV2 cells. We also found that TMC-256C1 activated p38 mitogen-activated protein kinases (MAPK) and phosphatidylinositol 3-kinase (PI3K)/Akt signaling pathways in HT22 and BV2 cells. These results demonstrated that TMC-256C1 activates HO-1 protein expression, probably by increasing nuclear Nrf2 levels via the activation of the p38 MAPK and PI3K/Akt pathways.

## 1. Introduction

Oxidative stress and neuroinflammation are associated with disorders of the central nervous system (CNS) [1]. Oxidative stress overexpression of reactive oxygen species (ROS) can induce neural injury and thus contribute to the progression of neurodegenerative diseases such as Alzheimer’s disease (AD) and Parkinson’s disease (PD), resulting in neuronal death and malfunction [2].

Glutamate, a main neurotransmitter in the CNS, plays a critical role in neurodegenerative diseases such as AD, PD, and Huntington’s disease [3]. Many studies have been conducted on glutamate-induced cytotoxicity in HT22 cell lines, an immortalized mouse hippocampal cell line lacking ionotropic glutamate receptors [4]. Neuroinflammation is a crucial mechanism underlying neuronal damage within the brain in neurodegenerative diseases including AD, PD, and amyotrophic lateral sclerosis [5,6]. Microglia, considered as macrophage in the brain, play a crucial role in immune defense in the central nervous system (CNS). Once stimulated, microglia produce inflammatory mediators including nitric oxide (NO), tumor necrosis factor-α (TNF-α), and prostaglandin E_2_ (PGE_2_) [7,8]. The BV2 cell line is considered to represent brain macrophages and is mainly used as a model of neuroinflammation [9,10,11]. In this study, mouse hippocampal HT22 cells and BV2 cells were used to examine the neuroprotective and anti-neuroinflammatory effects of certain secondary metabolites from nature.

Heme oxygenase (HO)-1 is a stress-responsive enzyme induced by stimulants including heat shock, oxidants and heavy metals. HO-1 plays a role as a catalyst for the degradation of heme groups, producing carbon monoxide, biliverdin, and free iron [12,13]. Heme and these metabolites play important roles in the regulation of several biological responses such as oxidative damage, cell injury, and inflammation. According to its anti-oxidative effects, HO-1 is recognized as a target for the therapy of inflammatory diseases [14,15]. It has been reported that HO-1 provides the anti-inflammatory action by suppressing the production of pro-inflammatory cytokines and chemokines such as TNF-α, IL-1β, IL-6, and MIP-1β in activated macrophages. In addition, HO-1 and its by-products suppress the expression of pro-inflammatory mediators such as inducible nitric oxide synthase (iNOS) and cyclooxygenase-2 (COX-2), leading to decreases in the production of NO and PGE_2_ [16,17].

The expression of HO-1 is regulated by nuclear transcription factor erythroid-2 related factor 2 (Nrf2), a main regulator of detoxifying/antioxidant phase II enzymes such as HO-1. Nrf2 binds to specific DNA sequences such as the anti-oxidant response element present in the promoter regions of phase II enzymes, and increases their transcription [18]. Recently, natural products such as curcumin, resveratrol, and flavonoids have been reported to show protective effects on neuronal damage induced by oxidative stress through the induction of HO-1 [19,20,21].

Marine-derived fungi have been regarded as promising sources of metabolites with diverse structures and bioactivities, and some metabolites from marine fungi have inspired the development of new classes of drugs [22,23,24]. In the course of our ongoing search for bioactive secondary metabolites from marine-derived fungi, TMC-256C1, a napthopyranone derivative [25] was isolated from marine-derived fungal strain *Aspergillus* sp. SF6354, and was found to exhibit cytoprotective and anti-inflammatory effects.

In this report, the isolation, structure elucidation, and bioactivity of TMC-256C1 are presented. Furthermore, the molecular mechanisms involved in the cytoprotective and anti-neuroinflammatory effects of TMC-256C1 were investigated.

## 2. Results

### 2.1. Structure Determination and Isolation of TMC-256C1, and Effect of TMC-256C1 on the Viability of HT22 and BV2 Cells

**Figure 1:**
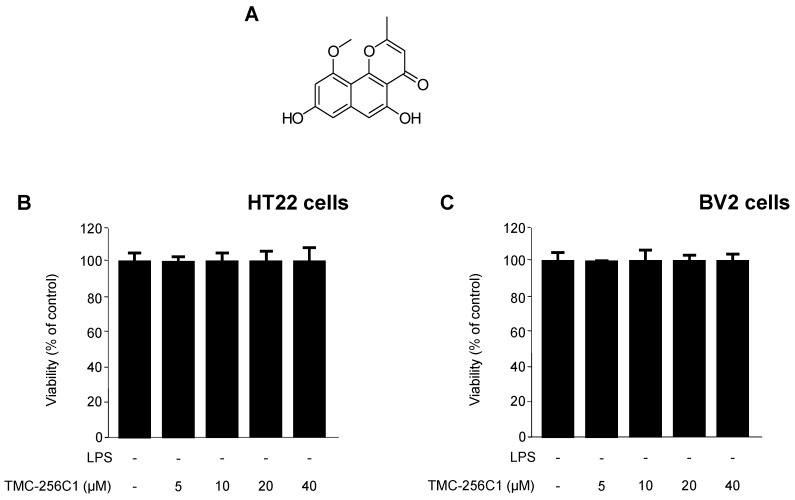
Chemical structure of TMC-256C1 (**A**); and effect of TMC-256C1 on cell viability (**B**,**C**). HT22 and BV2 cells were incubated for 24 h with various concentrations of TMC-256C1 (5–40 μM). Cell viability was determined as described in the Experimental Section. Bar represents the mean ± standard deviation (SD) of three independent experiments.

To identify the bioactive TMC-256C1 in the organic extract of culture media of the marine fungus *Aspergillus* sp. SF-6354, several purification methods were employed, including flash column chromatography and high performance liquid chromatography (HPLC), leading to the isolation of a napthopyrone fungal metabolite, TMC-256C1 (Figure 1A). The structure of the isolated TMC-256C1 was identified by analysis of nuclear magnetic resonance spectroscopy (NMR) and mass spectrometry (MS) data, along with comparison of its spectral data to those reported in the literature [26]. To evaluate the cytotoxic effect of TMC-256C1, we evaluated its effect on the viability of BV2 (Figure 1B) and HT22 cells (Figure 1C). MTT assay performed at 5–40 μM of TMC-256C1 revealed no cytotoxic effects in either HT22 or BV2 cells.

### 2.2. Effects of TMC-256C1 on Glutamate-Induced Cytotoxicity and Reactive Oxygen Species Generation

**Figure 2:**
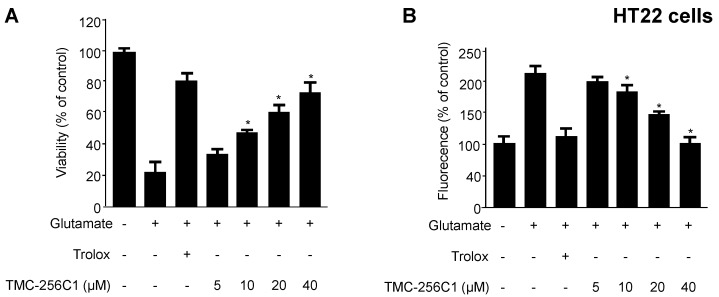
Effects of TMC-256C1 on glutamate-induced oxidative neurotoxicity (**A**); and generation of reactive oxygen species (**B**). HT22 cells were treated with TMC-256C1 and then incubated for 12 h with glutamate (5 mM) (**A**). Exposure of HT22 cells to glutamate resulted in increased reactive oxygen species production (**B**). Data are presented as the mean value of three experiments ± SD. * *p* < 0.05 compared to the group treated with glutamate. Trolox^®^ (50 μM) was used as a positive control.

At non cytotoxic concentrations (5–40 μM), we examined whether TMC-256C1 exerted cytoprotective effects and reactive oxygen species (ROS) scavenging activity against glutamate-induced cytotoxicity and ROS generation by treating HT22 cells with glutamate (5 mM) for 12 h (Figure 2A,B). Application of glutamate to the cells led to increased ROS production; however, pre-incubation (3 h) with TMC-256C1 effectively suppressed this induction (Figure 2B). Trolox^®^ is a well-known anti-oxidative agent, and was used as a positive control, showing a significant cytoprotective effect and ROS scavenging activity at the concentration of 40 μM.

### 2.3. Effects of TMC-256C1 on Pro-Inflammatory Cytokines in BV2 Cells Stimulated with Lipopolysaccharides (LPS)

**Figure 3:**
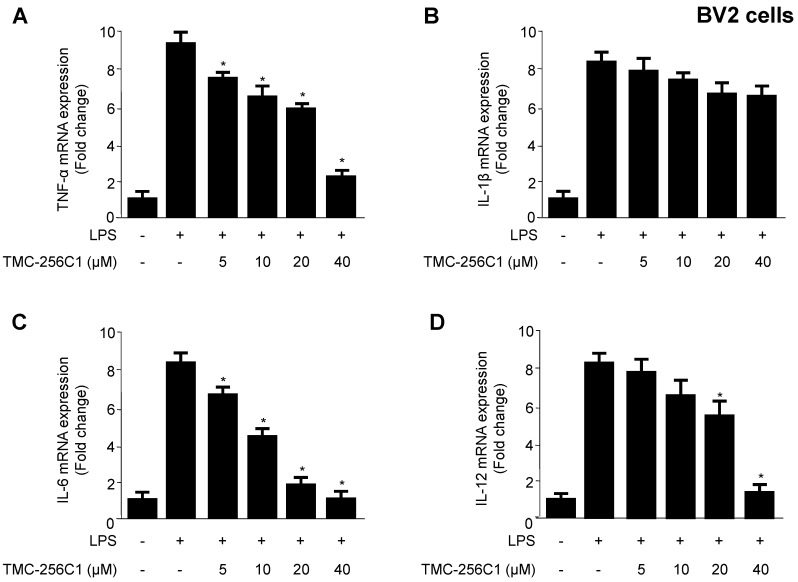
Effects of TMC-256C1 on mRNA expression levels of TNF-α (**A**); IL-1β (**B**); IL-6 (**C**); and IL-12 (**D**) in BV2 cells stimulated with lipopolysaccharide (LPS). Cells were pre-treated for 3 h with the indicated concentrations of TMC-256C1, and then stimulated for 12 h with LPS (1 μg/mL). The concentrations of TNF-α (**A**); IL-1β (**B**); IL-6 (**C**); and IL-12 (**D**) were determined as described in the Experimental Section. Data represent the mean values of three experiments ± SD. * *p* < 0.05 compared to the group treated with LPS.

To further evaluate the anti-neuroinflammatory effects of TMC-256C1 on lipopolysaccharides (LPS)-stimulated BV2 cells, the mRNA expression levels of pro-inflammatory cytokines such as TNF-α, IL-1β, IL-6 and IL-12 were evaluated in the both presence and absence of TMC-256C1 at non-cytotoxic concentrations (5–40 µM). BV2 cells were pretreated with TMC-256C1 for 3 h, followed by stimulation with LPS (1 µg/mL) for 12 h. As shown in Figure 3, LPS treatment triggered a significant increase in the mRNA expression levels of pro-inflammatory cytokines in the culture media, compared to those of the untreated group. However, pre-treatment of the cells with TMC-256C1 for 3 h significantly decreased the mRNA expression levels of TNF-α, IL-6 and IL-12 in a dose-dependent manner, but had little or no inhibitory effect on the mRNA expression of IL-1β.

### 2.4. Effects of TMC-256C1 on the Protein Expression Levels of Inducible Nitric Oxide Synthase (iNOS) and Cyclooxygenase-2 (COX-2) in BV2 Cells Stimulated with LPS

**Figure 4:**
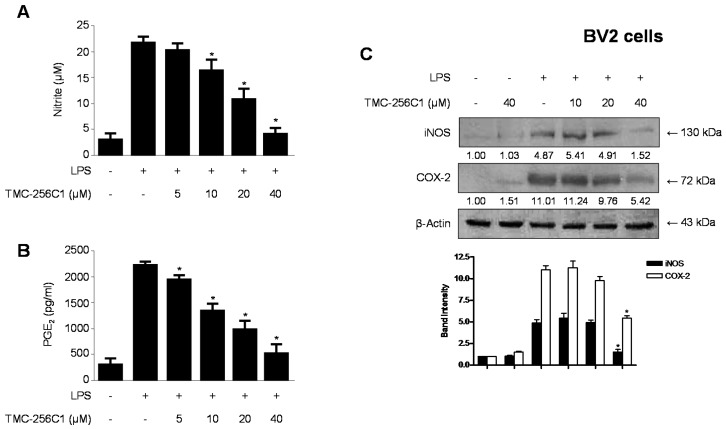
Effects of TMC-256C1 on the production of NO (**A**) and PGE_2_ (**B**) and the protein expression levels of inducible nitric oxide synthase (iNOS) and cyclooxygenase-2 (COX-2) (**C**) in BV2 cells stimulated with LPS. Cells were pre-treated for 3 h with the indicated concentrations of TMC-256C1, and for 24 h with LPS (1 μg/mL). Western blot analysis (**C**) was performed as described in the Experimental Section. Band intensity was quantified by densitometry and normalized to β-actin, and the normalized values are presented at the bottom of each band. Data represent the mean values of three experiments ± SD. * *p* < 0.05 compared to the group treated with LPS.

We also investigated the effects of TMC-256C1 on LPS-induced NO and PGE_2_ production as well as iNOS and COX-2 protein expression in BV2 cells (Figure 4). BV2 cells were challenged with LPS 1 μg/mL) in the presence or absence of TMC-256C1 at non-cytotoxic concentrations (5–40 µM). re-treatment of the cells with TMC-256C1 for 3 h resulted in a decreased concentration of iNOS-derived NO (Figure 4A) and iNOS expression (Figure 4C). Under the same conditions, TMC-256C1 also suppressed COX-2 expression (Figure 4C) and was also found to reduce COX-2-derived PGE_2_ production (Figure 4B). Our results showed that TMC-256C1 suppressed the LPS-induced production of inflammatory mediators (*i.e.*, NO and PGE_2_) via suppression of iNOS and COX-2 protein expression.

### 2.5. Effects of TMC-256C1 on NF-κB Activation in BV2 Cells Stimulated with LPS

**Figure 5:**
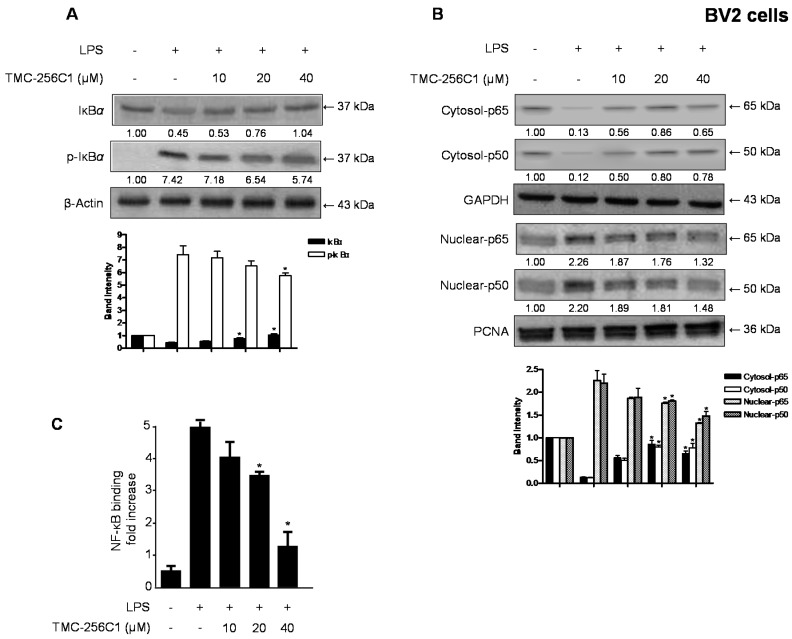
Effects of TMC-256C1 on LPS-induced NF-κB activation (**A**–**C**). Following pretreatment with TMC-256C1 (10, 20, and 40 μM) for 3 h, cells were treated with LPS for 1 h. Total proteins were prepared and Western blot analysis was performed using antibodies specific for IκB-α and p-IκB-α (**A**); Cytosolic and nuclear extracts were prepared for use in Western blots for NF-κB p65 and p50, using specific anti-p65 and anti-p50 monoclonal antibodies (**B**); A commercially available NF-κB ELISA (Active Motif) was used to test the nuclear extracts and determine the degree of NF-κB binding (**C**). Band intensity was quantified by densitometry and normalized to β-actin, glyceraldehyde-3-phosphate dehydrogenase (GAPDH) and proliferating cell nuclear antigen (PCNA), and the normalized values are presented at the bottom of each band. The data shown, representative of three independent experiments, are the mean values of three experiments ± SD. * *p* < 0.05 compared to the group treated with LPS.

Therefore, in order to elucidate the mechanisms underlying the anti-neuroinflammatory effects of TMC-256C1, we examined the effects of TMC-256C1 on the phosphorylation and degradation of IκB-α by Western blot analysis. As shown in Figure 5, IκB-α was phosphorylated and degraded after the cells were treated with LPS for 1 h, however, the phosphorylation and degradation of IκB-α were markedly suppressed by pre-treatment of the cells with TMC-256C1 (10–40 µM) for 3 h (Figure 5A). In accordance with this, the levels of nuclear NF-κB p65 and p50 in BV2 cells were increased after the cells were treated with LPS for 1 h, while the levels were decreased in response to pretreatment of TMC-256C1 (10–40 µM) in the LPS-stimulated cells in a dose-dependent manner (Figure 5B). In addition, LPS pre-treatment (1 h) led to an increased DNA-binding activity of NF-κB in nuclear extracts of BV2 cells; however, TMC-256C1 inhibited the DNA-binding activity of NF-κB in LPS-stimulated cells in a dose-dependent manner (Figure 5C). This observation suggested that TMC-256C1 inhibited the LPS-induced nuclear translocation of NF-κB (p65 and p50) through preventing the phosphorylation and degradation of IκB.

### 2.6. Effects of TMC-256C1 on HO-1 Expression in HT22 and BV2 Cells

**Figure 6:**
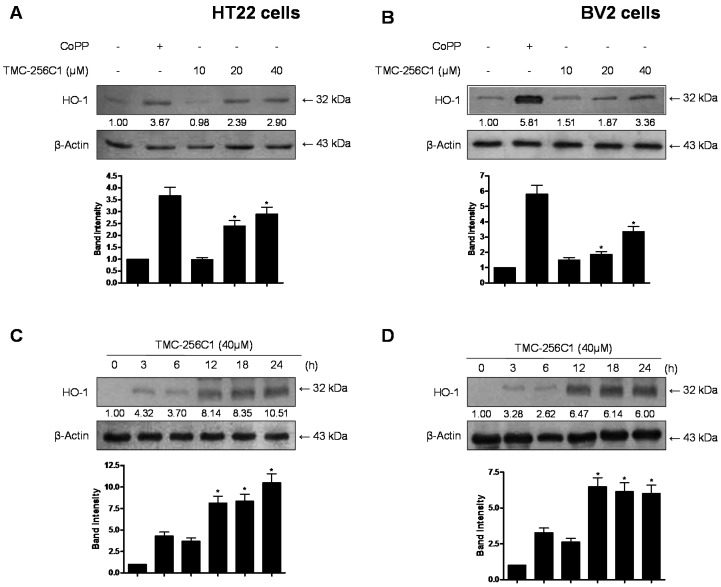
Effects of TMC-256C1 on hemeoxygenase (HO)-1 expression in HT22 cells (**A**,**C**) and BV2 cells (**B**,**D**). Cells were incubated for 12 h with the indicated concentrations of TMC-256C1 (**A**,**B**) and with 40 μM of TMC-256C1 (**C**,**D**). Western blot analysis for HO-1 expression was performed and representative blots of three independent experiments are shown. Band intensity was quantified by densitometry and normalized to β-actin, and the normalized values are presented at the bottom of each band. The data shown, representative of three independent experiments, are the mean values of three experiments ± SD. * *p* < 0.05 compared to the group treated with LPS.

To correlate the cytoprotective and anti-neuroinflammatory effects of TMC-256C1 with the induction of HO-1, we examined the effects of TMC-256C1 on HO-1 expression in HT22 and BV2 cells. Pre-treatment with TMC-256C1 at non-cytotoxic concentrations (10–40 µM) for 12 h dose-dependently increased HO-1 expression (Figure 6A,B) in HT22 and BV2 cells. The HO-1 inducer, cobalt protoporphyrin (CoPP) [27] was used as a positive control. The induction of HO-1 by TMC-256C1 reached a peak at 40 µM. At the concentration of 40 μM, HO-1 expression was first detected at 6 h, maximally increased at around 18 h, and then was gradually reduced after 24 h in HT22 and BV2 cells (Figure 6C,D).

### 2.7. Effects of HO-1 Expression on Glutamate-Induced Oxidative Neurotoxicity and the Inhibition of Pro-Inflammatory Mediators by TMC-256C1

**Figure 7:**
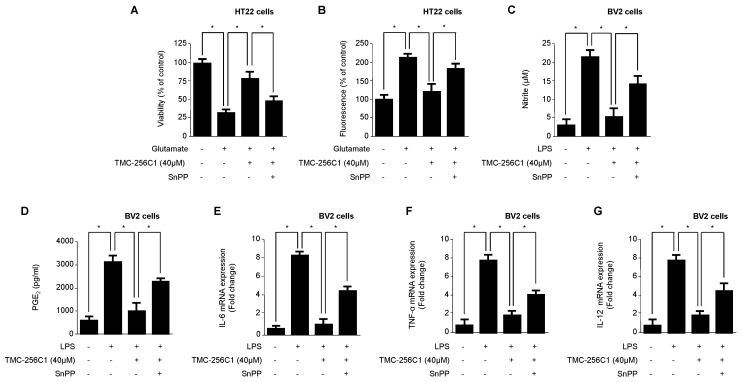
Effects of tin protoporphyrin (SnPP) on glutamate-induced oxidative neurotoxicity (**A**) and reactive oxygen species generation (**B**) in HT22 cells, and inhibition of nitrate (**C**); PGE_2_ (**D**); IL-6 (**E**); and TNF-α (**F**) IL-12 (**G**) production by TMC-256C1 pre-treatment of lipopolysaccharide (LPS)-stimulated BV2 cells. HT22 cells were pre-treated with TMC-256C1 in the presence or absence of SnPP (50 μM) and then incubated for 12 h with glutamate (5 mM) (**A**). Exposure of HT22 cells to 5 mM glutamate for 12 h in the presence or absence of SnPP (50 μM) increased reactive oxygen species production (**B**). BV2 cells were pre-treated for 3 h with TMC-256C1 (40 μM) in the presence or absence of SnPP (50 μM) and stimulated for 24 h with LPS (1 μg/mL) (**C**–**G**). HT22 cells and BV2 microglia were pretreated with SnPP for 3 h in this experiment. Data are presented as the mean value of three experiments ± SD. * *p* < 0.05 (Newman-Keuls *post hoc* test).

To confirm whether the cytoprotective effect of TMC-256C1 in HT22 cells and its anti-inflammatory effects in BV2 cells were mediated through the induction of HO-1 expression, the effect of SnPP, an inhibitor of HO-1, in TMC-256C1-treated cells was examined. HT22 cells were treated with 40 μM of TMC-256C1 for 12 h in the absence or presence of SnPP, and it was shown that SnPP significantly reversed the cytoprotective effect mediated by TMC-256C1 (Figure 7A). It was also shown that the induction of HO-1 expression by TMC-256C1 was necessary for suppressing the generation of glutamate-induced ROS (Figure 7B). Similarly, the potential correlation of HO-1 induction by TMC-256C1 with its anti-inflammatory effect was examined using SnPP. As shown in Figure 7C–G, pre-treatment with TMC-256C1 alone led to the inhibition of NO, PGE_2_, TNF-α, IL-6 and IL-12 production in LPS-stimulated cells. However, pre-treatment with both TMC-256C1 and SnPP partially reversed the inhibitory effects of TMC-256C1 on the production of those factors in LPS-stimulated cells.

### 2.8. Effects of TMC-256C1 on the Nuclear Translocation of Nrf2 in HT22 and BV2 Cells

**Figure 8:**
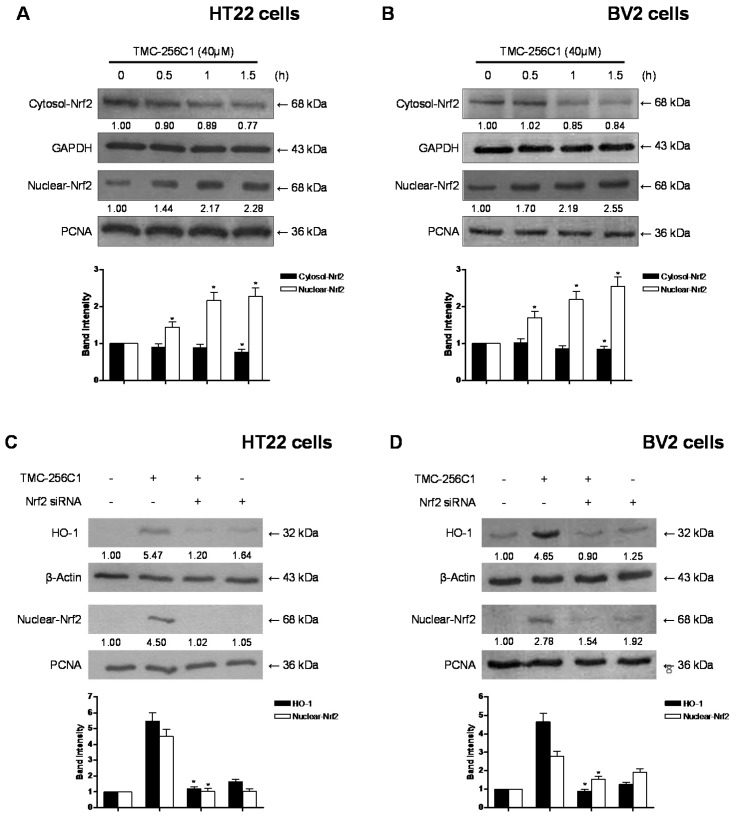
Effects of TMC-256C1 on the nuclear translocation of nuclear transcription factor-E2-related factor 2 (Nrf2) (**A**,**B**) and Nrf2-mediated HO-1 (**C**,**D**) in HT22 cells and BV2 cells. Cells were treated with 40 μM of TMC-256C1 for 0.5, 1, and 1.5 h (**A**,**B**). The nuclei were fractionated from the cytosol using M-PER™ Mammalian Protein Extraction buffer (Pierce Biotechnology, Rockford, IL, USA). HT22 cells and BV2 cells were transiently transfected with Nrf2 siRNA and then treated with 40 μM TMC-256C1 for 12 h (**C**,**D**). Nrf2 protein was detected by Western blot analysis and representative blots of three independent experiments are shown. Band intensity was quantified by densitometry and normalized to β-actin, glyceraldehyde-3-phosphate dehydrogenase (GAPDH) and proliferating cell nuclear antigen (PCNA), and the normalized values are presented at the bottom of each band. The data shown, representative of three independent experiments, are the mean values of three experiments ± SD. * *p* < 0.05 compared to the group treated with LPS.

Nuclear translocation of activated Nrf2 is an important upstream contributor to the transcription of HO-1 [28]. Therefore, we investigated whether treatment of HT22 and BV2 cells with TMC-256C1 induced nuclear translocation of Nrf2 to the cell nuclei. The cells were incubated with TMC-256C1 for 0.5, 1 and 1.5 h at a concentration of 40 μM, and the level of Nrf2 protein was then determined by western blot analysis. As shown in Figure 8A,B, the protein levels of Nrf2 in the nuclear fractions of TMC-256C1-treated cells were gradually increased compared with those of the untreated cells, while they were concomitantly decreased in the cytoplasmic fractions. Moreover, the role of Nrf2 in HO-1 expression by TMC-256C1 was studied using siRNA against Nrf2. HT22 and BV2 cells were transiently transfected with siRNA Nrf2 and then were treated with TMC-256C1 for 12 h to induce HO-1 expression. As shown in Figure 8C,D, Nrf2 siRNA have completely blocked off nuclear translocation of Nrf2. In addition, transient transfection with Nrf2 siRNA also abolishes induction of HO-1 expression by TMC-256C1 in both HT22 and BV2 cells. These results indicated that HO-1 induction upon incubation with TMC-256C1 is related to the Nrf2 nuclear translocation pathway in HT22 and BV2 cells.

### 2.9. Involvement of the Mitogen-Activated Protein Kinases (MAPK) Pathways in the Induction of HO-1 Expression by TMC-256C1

**Figure 9:**
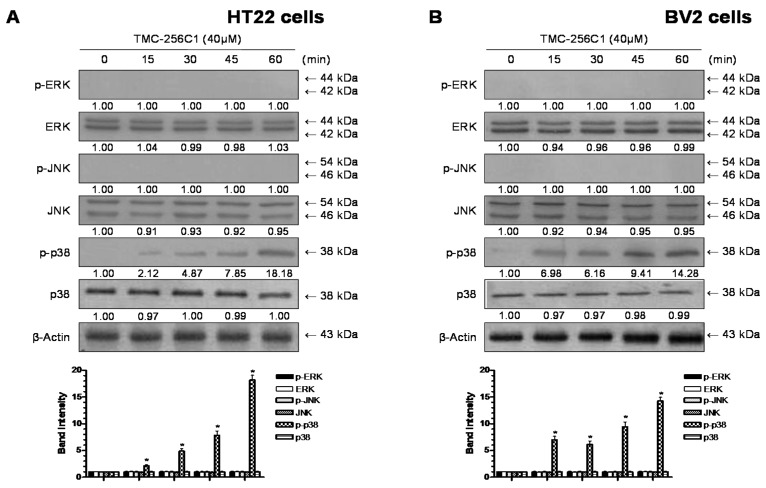
Effects of TMC-256C1 on extracellular signal-regulated kinases (ERK), c-Jun N-terminal kinase (JNK) and p38 mitogen-activated protein kinases (MAPK) expression in HT22 cells (**A**) and BV2 cells (**B**). Cells were treated with 40 µM TMC-256C1 for the indicated times. Cell extracts were analyzed by Western blot with antibodies specific for phosphorylated ERK1/2 (p-ERK), phosphorylated JNK (p-JNK), or phosphorylated p38 (p-p38). Membranes were stripped and re-probed using antibodies with affinity for both the phosphorylated and non-phosphorylated forms of each MAPK as a control, and the representative blots of three independent experiments are shown. Band intensity was quantified by densitometry and normalized to β-actin, and the normalized values are presented at the bottom of each band. The data shown, representative of three independent experiments, are the mean values of three experiments ± SD. * *p* < 0.05 compared to the group treated with LPS.

**Figure 10:**
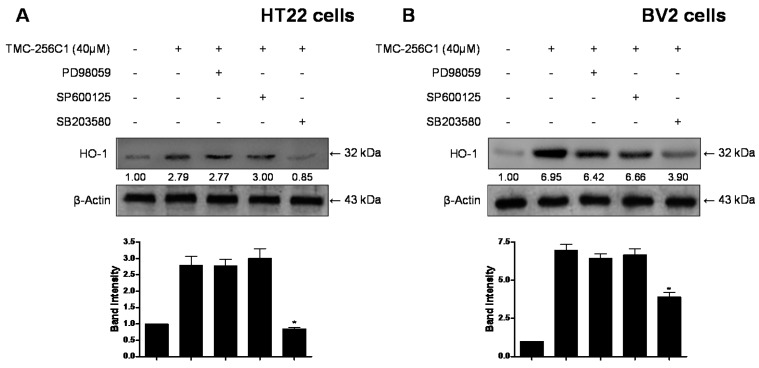
Effects of p38 activation induced by TMC-256C1 on HO-1 expression in HT22 cells (**A**) and BV2 cells (**B**). Cells were pre-treated for 1 h with the specific inhibitors, PD98059 (40 μM), SP600125 (25 μM), and SB203580 (20 μM), and then treated with TMC-256C1 (40 μM) for 12 h. Band intensity was quantified by densitometry and normalized to β-actin, and the normalized values are presented at the bottom of each band. The data shown, representative of three independent experiments, are the mean values of three experiments ± SD. * *p* < 0.05 compared to the group treated with LPS.

Mitogen-activated protein kinases (MAPK) is activated in response to oxidative stress and other various stress factors. It has been reported that the activation of the MAPK pathways are involved in the induction of HO-1 [29]. Therefore, to identify the signaling pathways involved in the induction of HO-1 expression by TMC-256C1, we analyzed its effects on the three MAPK pathways. The activation of these pathways was analyzed with activation-specific antibodies that selectively recognize the active and phosphorylated forms of extracellular signal-regulated kinases (ERK), c-Jun N-terminal kinase (JNK), and p38. At the concentration of 40 μM, TMC-256C1 activated the p38 pathway by increasing the phosphorylation of p38 in both HT22 and BV2 cells. Phosphorylation of p38 was observed 15 min after the treatment of cells with TMC-256C1, and was sustained for 60 min (Figure 9). On the other hand, the phosphorylation of JNK and ERK was not observed. Furthermore, to confirm the role of p38 kinase in regulating the induction of HO-1 expression by TMC-256C1 in BV2 and HT22 cells, the effects of specific inhibitors of ERK (PD98059), JNK (SP600125) and p38 (SB203580) on the expression levels of HO-1 were examined by Western blot analysis (Figure 10). The results indicated that HO-1 expression induced by TMC-256C1 was inhibited by the p38 inhibitor, whereas the JNK and ERK inhibitors had little or no effect on HO-1 protein levels. These results suggested that activation of the p38 pathway might be involved in the induction of HO-1 expression by TMC-256C1.

### 2.10. Involvement of the PI3K/Akt Pathway in the Induction of HO-1 Expression by TMC-256C1

**Figure 11:**
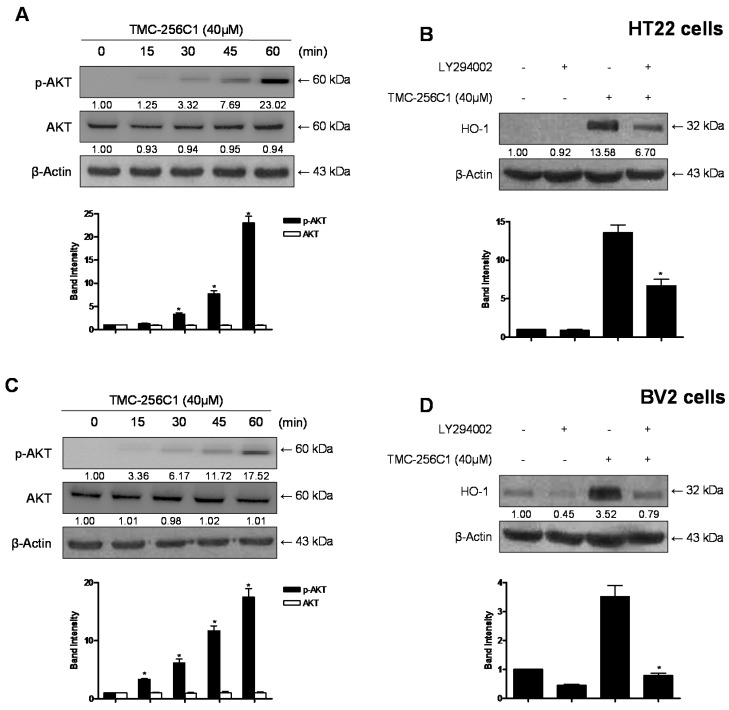
Effects of TMC-256C1 on HO-1 expression through the PI3K/AKT cascade in HT22 and (**A**,**B**) and BV2 cells(**C**,**D**). Cells were treated with TMC-256C1 (40 μM) for the indicated times (**A**,**C**). Cells were pre-incubated with or without 10 μM LY294002 for 1 h and then incubated in the absence or presence of 40 μM of TMC-256C1 for 12 h (**B**,**D**). Cell extracts were analyzed by Western blots with specific antibodies, and representative blots of three independent experiments are shown. Band intensity was quantified by densitometry and normalized to β-actin, and the normalized values are presented at the bottom of each band. The data shown, representative of three independent experiments, are the mean values of three experiments ± SD. * *p* < 0.05 compared to the group treated with LPS.

There have been several studies showing that the PI3K/Akt pathway is involved in the induction of HO-1 expression by plant metabolites [30,31,32]. Therefore, additional analysis was conducted to correlate the induction of HO-1 by TMC-256C1 with the activity of the PI3K/Akt pathway. When the cells were treated with 40 μM of TMC-256C1, the phosphorylation levels of Akt in the cells gradually increased from 15 to 60 min (Figure 11A,C). Furthermore, pre-treatment of the cells with LY294002 (a specific inhibitor of PI3K) attenuated the induction of HO-1 expression by TMC-256C1 (Figure 11B,D). These results suggested that TMC-256C1-induced expression of HO-1 was mediated through the PI3K/Akt pathway in HT22 and BV2 cells.

## 3. Discussion

Oxidative stress and neuroinflammation are known to influence the etiology of many [33]. Therefore, searching for natural products with both anti-oxidative and anti-inflammatory activities could be a promising approach for the treatment of various neurodegenerative diseases, and several natural products have previously been identified as antioxidant and anti-inflammatory agents with their effects on the regulation of MAPK and HO-1 [34,35,36,37].

In this study, it was shown that TMC-256C1 has a protective activity against glutamate-induced cytotoxicity in HT22 cells. TMC-256C1 suppressed glutamate-induced cell death and effectively inhibited the generation of ROS induced by glutamate in HT22 cells. Furthermore, it was demonstrated that the induction of HO-1 is closely related to the cytoprotective effect of TMC-256C1 against glutamate-induced oxidative stress in HT22 cells [38]. In this study, TMC-256C1 induced the expression of HO-1 in a dose-dependent manner, and this was necessary to suppress the generation of ROS by glutamate. Therefore, the cytoprotective effect of TMC-256C1 might be mediated through the induction of HO-1 expression.

Overproduction of inflammatory mediators by stimulated microglia may aggravate neuronal degeneration [39]. TMC-256C1 suppressed the gene expression of pro-inflammatory cytokines such as TNF-α, IL-6, and IL-12 in BV2 cells treated with LPS. TMC256-C1 also suppressed the production of the inflammatory mediators such as NO and PGE_2_ by down-regulating iNOS and COX-2 protein expression in the cells. NF-κB regulates the expression of a variety of genes including iNOS, COX-2, TNF-α, and IL-1β [40,41]. With respect to this pathway, TMC256-C1 was found to inhibit the phosphorylation and degradation of IκB-α as well as the DNA-binding activity of NF-κB in LPS-stimulated BV2 cells, indicating the correlation of its anti-inflammatory effects with the inhibition of the activation of the NF-κB pathway in LPS-stimulated BV2 cells. TMC-256C1 also increased HO-1 expression and activity in BV2 cells in a dose-dependent manner. The correlation between the induction and activation of HO-1 and the resulting anti-inflammatory action in microglia has been reported [14,16]. In a line with this, the inhibitory effects of TMC-256C1 on cell death and ROS production in glutamate-treated HT22 cells as well as the production of pro-inflammatory mediators and cytokines in LPS-stimulated BV2 cells were partially reversed by the treatment with SnPP, an inhibitor of HO-1 enzyme activity.

**Figure 12:**
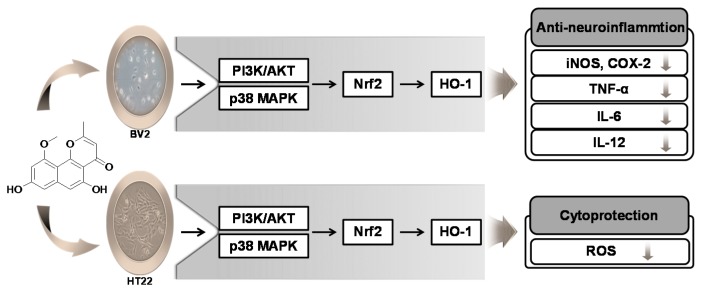
The suggested mechanism for the anti-neuroinflammatory and cytoprotective effects of TMC-256C1 in HT22 and BV2 cells. TMC-256C1 increased cellular resistance to oxidative injury induced by glutamate-induced oxidative cytotoxicity in HT22 cells, via Nrf2-dependent HO-1 expression. In BV2 cells, TMC-256C1 inhibited the LPS-induced production of pro-inflammatory mediators possibly through the Nrf2-dependent HO-1 expression. Especially, PI3K/Akt and p38 MAPK regulate the Nrf2 activation in both HT22 and BV2 cells.

The Nrf2 transcription factor plays a crucial role in the expression of phase 2 detoxifying and antioxidant enzymes and in the activation of other inducible genes by various stimuli in response to oxidative stress [42]. Nrf2 is required for the expression of some inducible proteins including glutathione S-transferase, quinine reductase, and HO-1 [16]. In the present study, it was shown that TMC-256C1 promoted the translocation of Nrf2 into the nucleus, suggesting that Nrf2 may play an essential role in the induction of HO-1 expression by TMC-256C1. MAPK and PI3K/Akt and their associated signaling pathways are also involved in the regulation of HO-1 gene expression through the phosphorylation and activation of the Nrf2 pathway (Figure 12) [43,44]. In this study, the activation of the p38 and PI3K/Akt pathways appeared to be involved in the induction of HO-1 expression by TMC256-C1.

## 4. Experimental Section

### 4.1. Instruments, Fungal Materials and Isolation of TMC-256C1

Electrospray ionization mass spectrometry (ESIMS) data were obtained using a quadrupole time of flight micro liquid chromatography-MS/MS instrument (Waters, Manchester, UK). NMR spectra were recorded in DMSO-*d*_6_ with a JNM ECP-400 spectrometer (JEOL, Peabody, MA, USA), and the chemical shifts were referenced relative to the residual solvent peaks (δ_H_/δ_C_ = 2.49/39.5). HPLC (YOUNGLIN-YL9100, Younglin, Anyang, Korea) separation was performed using a Synergi™ 4u Polar-RP 80A, AXIA™ packed column (column dimensions of 21.2 × 150 mm, 5 μm particle size; Phenomenex, Macclesfield, UK) with a flow rate of 5 mL/min. The solvents used for HPLC were all of analytical grade.

*Aspergillus* sp. SF-6354 (deposited at the College of Medical and Life Sciences fungal strain repository, Silla University, Busan, Korea) was isolated from an unidentified sponge collected in the Sea of Ross (S 78°28.728′, E 163°22.694′) at a depth of 375 m in February 2013. This fungus was identified based on ribosomal RNA (rRNA) sequence analysis. A GenBank search with the 28S rRNA gene of SF-6354 (GenBank accession number KT185660) indicated *Aspergillus niger* (KC119204), *A. heteromorphus* (KM434330), and *A. japonicus* (KC128815) as the closest matches, with sequence identities of 99.42%, 98.83%, and 96.85%, respectively. Therefore, the marine-derived fungal strain SF-6354 was classified as *Aspergillus* sp.

The fungal strain was cultured on 10 Petri dishes (90 mm diameter), each containing 20 mL of potato dextrose agar medium (0.4% (*w*/*v*) potato starch, 2% (*w*/*v*) dextrose, 3% (*w*/*v*) NaCl, and 1.5% (*w*/*v*) agar). The plates were individually inoculated with 2 mL of seed cultures of the fungal strain, and they were incubated at 25 °C for a period of 14 days. Extraction of the agar media with ethyl acetate (150 mL) provided an organic phase, which was then concentrated *in vacuo* to yield 563.5 mg of extract. The ethyl acetate extract was subjected to C18 flash column chromatography (column dimensions of 3.5 × 17.0 cm), eluting with a stepwise gradient of 20%, 40%, 60%, 80%, and 100% (*v*/*v*) methanol in H_2_O (250 mL of each solution). The fraction that eluted at 80% methanol (151.2 mg) was further separated by C18 functionalized column chromatography, eluting with 75% (*v*/*v*) methanol in H_2_O, yielding seven subfractions. Subfraction 4 was purified by semipreparative reversed-phase high-pressure liquid chromatography (HPLC), eluting with a gradient from 60% to 63% (*v*/*v*) methanol in H_2_O containing 0.1% (*v*/*v*) formic acid over 60 min, to yield TMC-256C1 (2.3 mg, *t*_R_ = 31.6 min, yield = 0.41% (*w*/*w*)).

TMC-256C1: pale yellow, HRESIMS: *m*/*z* 273.0764 [M + H]^+^ (calculated for C_15_H_13_O_5_, 273.0763) (Figure S1), ^1^H-NMR (400 MHz, CDCl_3_) δ: 12.88 (1H, s, 5-OH), 10.52 (1H, brs, 8-OH), 6.77 (1H, s, H-6), 6.61 (1H, d, *J* = 2.1 Hz, H-7), 6.47 (1H, d, *J* = 2.1 Hz, H-9), 6.45 (1H, s) 3.93 (3H, *s*, 10-OCH_3_), 2.47 (3H, s, 2-CH_3_) (Figure S2), ^13^C-NMR (100 MHz, CDCl_3_) δ: 182.1 (C-4), 167.5 (C-2), 160.1 (C-8), 159.0 (C-10), 155.5 (C-11), 155.4 (C-5), 140.7 (C-13), 109.7 (C-3), 107.4 (C-12), 104.2 (C-6), 103.0 (C-14), 101.1 (C-7), 97.4 (C-9), 55.9 (10-OCH_3_), 19.9 (2-CH_3_) (Figure S3).

### 4.2. Chemicals and Reagents

Dulbecco’s modified Eagle’s medium (DMEM), fetal bovine serum (FBS), and other tissue culture reagents were purchased from Gibco BRL Co. (Grand Island, NY, USA). Tin protoporphyrin IX (SnPP IX), an inhibitor of HO activity, was obtained from Porphyrin Products (Logan, UT, USA). All other chemicals were obtained from Sigma-Aldrich (St. Louis, MO, USA). Primary antibodies including mouse/goat/rabbit anti-HO-1, Nrf2, COX-2, iNOS, β-Actin, IкB-α, p-IкB-α, p50, p65, GAPDH and PCNA, and also secondary antibodies were purchased from Santa Cruz Biotechnology (Heidelberg, Germany), and p-ERK, ERK, p-JNK, JNK, p-p38, p38 p-AKT and AKT antibodies were obtained from Cell Signaling Technology (Cell Signaling, Danvers, MA, USA). Enzyme-linked immunosorbent assay (ELISA) kit for PGE_2_ was purchased from R&D Systems, Inc. (Minneapolis, MN, USA) [45].

### 4.3. Cell Culture and Viability Assay

HT22 and BV2 cells were maintained at a density of 5 × 10^5^ cells/mL in DMEM medium with supplement, and were incubated according to the method described previously [45,46].

### 4.4. Quantitative Reverse-Transcription Polymerase Chain Reaction

Total RNA was isolated from the cells using Trizol (Invitrogen, Carlsbad, CA, USA), in accordance with the manufacturer’s recommendations, and quantified spectrophotometrically at a wavelength of 260 nm. Total RNA (4 μg) was reverse-transcribed using a High Capacity RNA-to-cDNA kit (Applied Biosystems, Carlsbad, CA, USA). The cDNA was then amplified using a SYBR Premix Ex Taq kit (TaKaRa Bio Inc., Shiga, Japan) and a StepOnePlus Real-Time PCR system (Applied Biosystems). The primer sequences were as follows: 5′-CCA GAC CCT CAC ACT CAC AA-3′ and 5′-ACA AGG TAC AAC CCA TCG GC-3′ forward and reverse primers for TNF-α, 5′-AAT TGG TCA TAG CCC GCA CT-3′ and 5′-AAG CAA TGT GCT GGT GCT TC-3′ forward and reverse primers for IL-1β, 5′-TCA CAA GTC GGA GGC TT-3′ and 5′-TGC AAG TGC ATC ATC GTT GT-3′ forward and reverse primers for IL-6, and 5′-AGT GAC ATG TGG AAT GGC GT-3′ and 5′-CAG TTC AAT GGG CAG GGT CT-3′ forward and reverse primers for IL-12 [45].

### 4.5. DNA-Binding Activity of NF-κB

The DNA-binding activity of NF-κB in nuclear extracts was measured using a TransAM kit (Active Motif, Carlsbad, CA, USA) according to the manufacturer’s instructions as described previously [47].

### 4.6. Preparation of Cytosolic and Nuclear Fractions

Cells were homogenized (1:20, *w*/*v*) in PER-Mammalian Protein Extraction buffer (Pierce Biotechnology, Rockford, IL, USA) containing freshly added protease inhibitor cocktail I (EMD Biosciences, San Diego, CA, USA) and 1 mM phenyl methyl sulfonyl fluoride. The details of procedures for the preparation of nuclear and cytosolic fractions are described elsewhere [46].

### 4.7. Nitrite (NO Production) and PGE_2_ Determination

The production of nitrite in conditioned media was determined according to the method described previously [46]. Levels of PGE_2_ present in each sample were determined using a commercially available kit from R&D Systems. The assay was performed according to the manufacturer’s instructions as described previously [46].

### 4.8. Western Blot Analysis

Cells were harvested by centrifugation at 200× *g* for 3 min. Subsequently, the cells were washed with phosphate buffered saline (PBS) and used by the radioimmunoprecipitation assay (RIPA) buffer containing 25 mM Tris-HCl buffer (pH 7.6) (Pierce Biotechnology, Rockford, IL, USA), 150 mM NaCl, 1% NP-40, 1% sodium deoxycholate, and 0.1% SDS. The details of Western blot analysis are described elsewhere [45].

### 4.9. Reactive Oxygen Species Measurement

The measurement of reactive oxygen species was determined according to the method described previously [48]. Briefly, HT22 cells (2.5 × 10^4^ cells/well in 24-well plates) were treated with 5 mM glutamate in the presence or absence of TMC-256C1 or SnPP (HO inhibitor) and incubated for 12 h [48].

### 4.10. Statistical Analysis

The data are expressed as the mean ± standard deviation (SD) of at least 3 independent experiments. To compare three or more groups, one-way analysis of the variance was used, followed by Tukey’s multiple comparison tests. The statistical analysis was performed with GraphPad Prism software, version 3.03 (GraphPad Software Inc., San Diego, CA, USA) [45].

## 5. Conclusions

TMC-256C1, a metabolite isolated from the marine-derived fungus *Aspergillus* sp., was discovered to have neuroprotective and anti-neuroinflammatory effects. TMC-256C1 was originally identified as an inhibitor of IL-4 signal transduction [25]. However, the neuroprotective and anti-neuroinflammatory activities of this metabolite have not previously been reported. This study demonstrated that TMC-256C1 possesses these activities, and that they are mediated through the induction of HO-1 expression via the Nrf2 pathway. In addition, the PI3K/Akt and p38 pathways were suggested to be involved in activation of the Nrf2 pathway by TMC-256C1.

## Supplementary Materials

Supplementary materials can be found at http://www.mdpi.com/1422-0067/17/4/529/s1.
