# Supplementary material for: Anti-Inflammatory and Cytoprotective Effects of TMC-256C1 from Marine-Derived Fungus Aspergillus sp. SF-6354 via up-Regulation of Heme Oxygenase-1 in Murine Hippocampal and Microglial Cell Lines
Source: Int J Mol Sci. 2016 Apr 8;17(4):529. doi: 10.3390/ijms17040529 (PMC4848985; doi:10.3390/ijms17040529)
Supplement: Supplementary file 1 [file ijms-17-00529-s001.pdf]

Supplementary Materials: Anti-Inflammatory and Cytoprotective Effects of TMC-256C1 from Marine-Derived Fungus *Aspergillus* sp. SF-6354 via up-Regulation of Heme Oxygenase-1 in Murine Hippocampal and Microglial Cell Lines

Dong-Cheol Kim, Kwang-Ho Cho, Wonmin Ko, Chi-Su Yoon, Jae Hak Sohn, Joung Han Yim, Youn-Chul Kim and Hyuncheol Oh

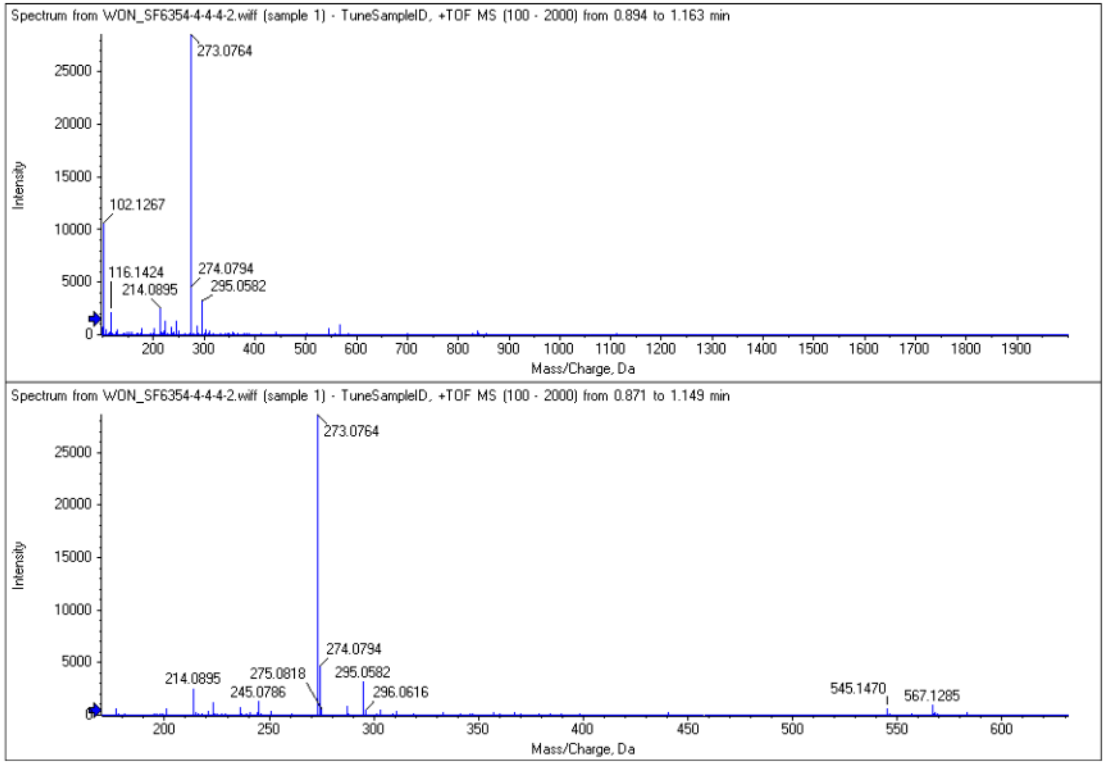

Figure S1. Electrospray ionization-high resolution time-of-flight (HRESITOF) mass spectrum of **1**.

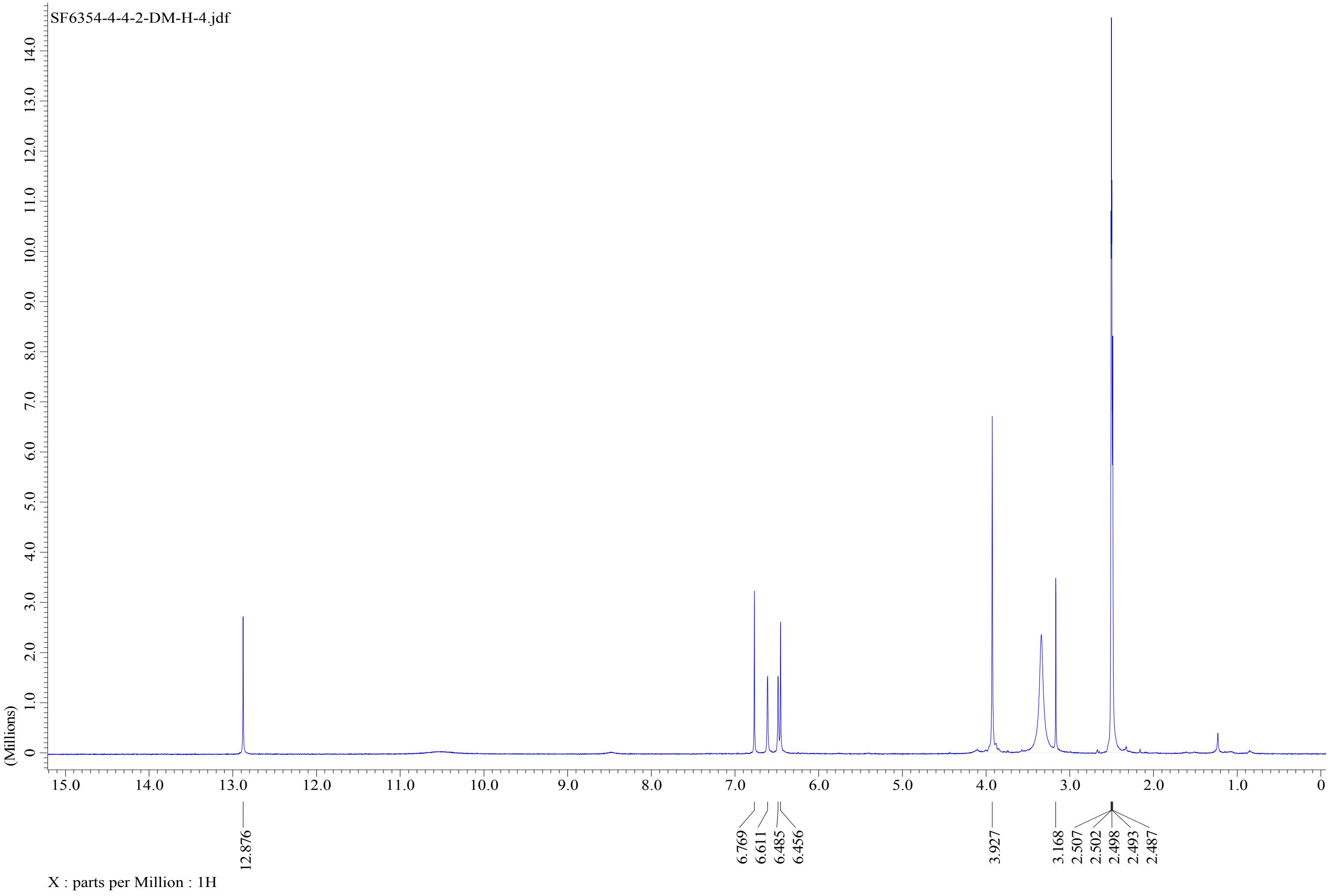

Figure S2. ^{1}H-NMR spectrum of TMC-256C1 (400 MHz, DMSO-d_6).

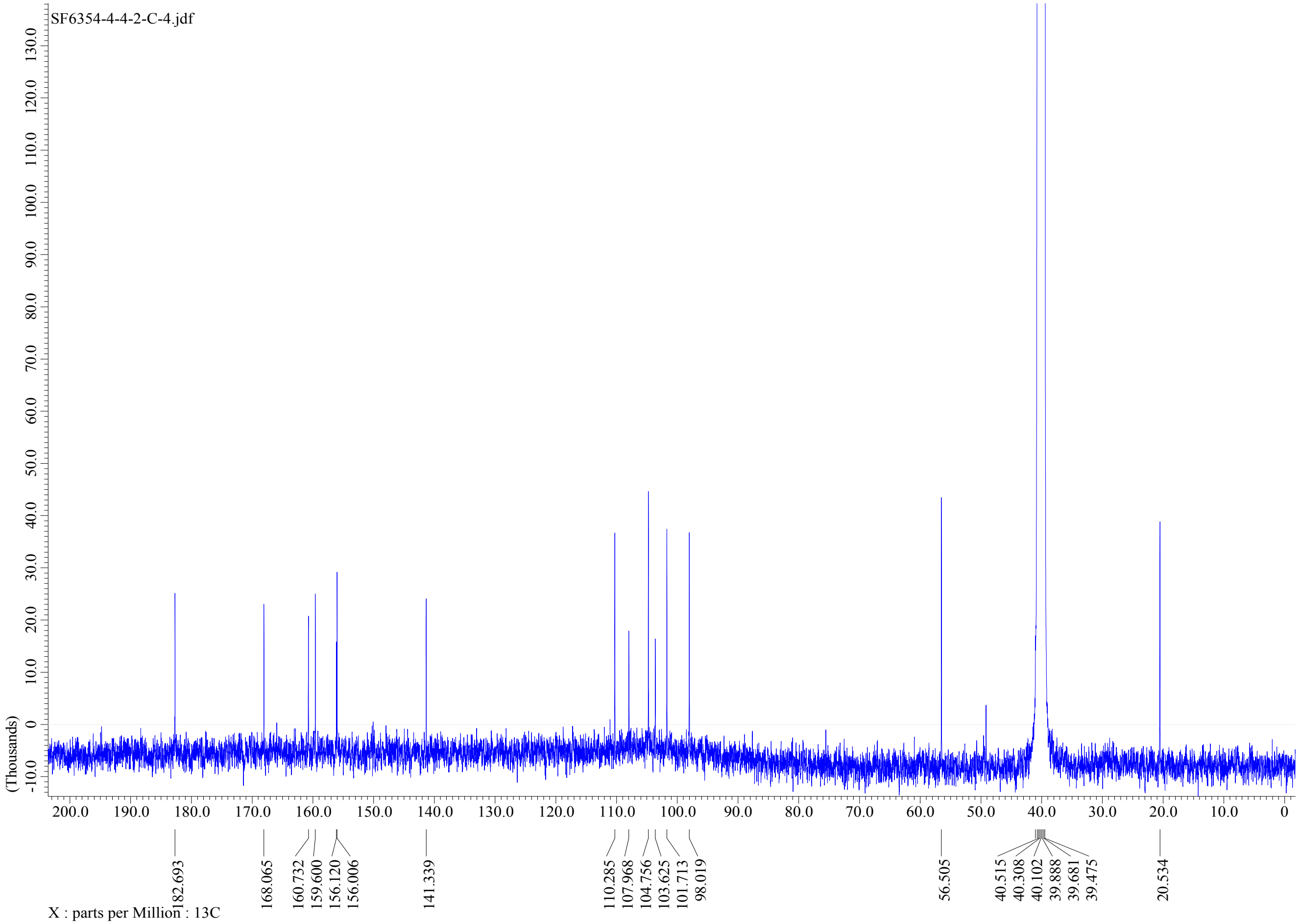

Figure S3. ^{13}C-NMR spectrum of TMC-256C1 (100 MHz, DMSO-d_6).
